# Supplementary material for: Utilization of companionship during delivery and associated factors among women who gave birth at Arba Minch town public health facilities, southern Ethiopia
Source: PLoS One. 2020 Oct 2;15(10):e0240239. doi: 10.1371/journal.pone.0240239 (PMC7531811; doi:10.1371/journal.pone.0240239)
Supplement: S1 File — (DOCX) [file pone.0240239.s001.docx]

**Questionnaire in English version**

Questionnaire ID._____________________________

Part I .Socio demographic

| 101 | How old are you? | Age in years_________ |
| --- | --- | --- |
| 102 | Where do you live? | 1. Rural  2. Urban |
| 103 | What is your Current marital status? | 1. Married  2. single  3. Divorced  4. widowed |
| 104 | Religion | 1.orthodox  2.protestant  3.muslim  4.catholic  5. others |
| 105 | Ethnicity | 1.Gamo  2.Gofa  3. Welayta  4.Amhara  5.Oromo  6.others(specify)_______ |
| 106 | What is your level of education? | 1. 1. Not read and write 2. 2. Primary school   3. Secondary school  4.above Secondary |
| 107 | What is your Occupational status? | 1. 1. House wife 2. 2. Government employer 3. 3.marchent/private 4. 4. NGO 5. 5. others (specify)__________ |
| 108 | What is your husband level of education? | 1. Not read and write  2. Primary school  3. Secondary school  4.above Secondary |
| 109 | Monthly family income? | --------in birr |

**B.** Utilization of companionship during childbirth.

|  | Questions | Coding category | Skip |
| --- | --- | --- | --- |
| 201 | Do you have support person during this labor? | 1. 1. yes 2. 2. no | If no ,skip to no 201b |
| 201a | If yes, who was the support person? | 1.Husband  2.Sister/brother/in law  3.Mother/mother in law  4.Freinds/neighbors   1. 5. Others(specify) |  |
| 201b | If no, why? | 1 .I didn't want  2. institution not allow  3. providers not allow  4. I have no support   1. 5. others------------ |  |
| 202 | Do you have support person during delivery? | 1. Yes 2. no |  |
| 202a | If yes, who was the support person? | 1.Husband  2.Sister  3.Mother  4.Freinds  5. Others(specify) |  |
| 202b | If no support, why? | 1 .I didn't want  2. institution not allow  3. providers not allow  4. I have no support  5. cesarean delivery |  |
| 203 | Route of delivery | 1.vaginal 2.cesarean |  |

**C: Knowledge and desire of women companionship**

| 204 | Do you know what labor companion is? | 1.yes 2.no | If no, skip to 205 | |
| --- | --- | --- | --- | --- |
| 204a | If yes, what it mean by | 1. support during labor  2. support during pregnancy  3. support after delivery  4. others |  | |
| 205 | Do you think that labor companion has positive birth outcome? | 1. Yes  2. No |  | |
| 206 | Do you know that every woman has the right to have companionship while she is in labor? |  | If no, skip to 207 | |
| 206a | If yes, where do you get this information? | 1.heard from people  2.I experienced it before  3.from health providers(ANC)  4. I read about it  5. others(specify) |  | |
| 207 | What do you say about having support person during labor? | 1. Good practice  2. not good |  | |
| 208 | Do you expect any help from companion? | 1. Yes 2. No | If no, skip to 209 | |
| 208a | If yes, what would be your expectations from companion to do for you in labor?  For each expectation 1 or 2  1=yes  2=no | 1.Encourage  2.rub your back  3. pray for me  4.cover medical expense  5.others (specify) |  | |
| 209 | Do you think that having labor companionship is beneficial? | 1. Yes  2. No | | If no skip to q210 |
|  |  |  |  |  |
| 209a | If yes what are the benefits | 1.reduced pain in labor | |  |
|  |  | 2.reduced need for c/s | |  |
|  |  | 3.increased chance of VD | |  |
|  |  | 4.reduced worry & fear | |  |
|  |  | 5.Make the women happy | |  |
|  |  | 6.reduce the duration of labor | |  |
|  |  | 7.reduced loneliness in women | |  |
|  |  | 8. Survival of baby is better | |  |
|  |  | 9.reduce the chance of abuse & disrespect of women by health care providers | |  |
|  |  | 10. others(specify)---- | |  |
|  | **Desire to have companion during delivery** |  | |  |
| 210 | Did you wish to have companion during delivery? | 1. Yes 2. No | | If no skip to D |
| 210a | If not, the reason for not desiring to have companionship? | 1.not to exposed 2.to be alone 3.others------------- | |  |

**D: Obstetric (past and present) data**

| Q.no. | Questions | Choices for response | Skip | |  |  |
| --- | --- | --- | --- | --- | --- | --- |
| 301 | How many times have you delivered a baby/ies before? | ______ in numbers | If no skip to q 302 | |  |  |
| 301a | If multiparous, where did you deliver your previous baby? | 1.at home  2.at health center  3. at hospital  4. at private facility  5. others ----- |  | |  |  |
| 301b | If you deliver at home, why? | 1.to be attended by TBAs |  | | |  |
|  |  | 2.my pregnancy is normal |  | | | |
|  |  |  |  | | | |
|  |  | 3.fear of being abused & disrespected |  |  |  |  |
|  |  | 4.it is usual practice |  | | | |
|  |  | 5.fear of being lonely in labor |  | | | |
|  |  | 6.short labor duration |  | | | |
|  |  | 7.no transport |  | | | |
|  |  | 8.others(specify)_______ |  | | | |
|  |  |  |  |  |  |  |

| 302 | Did you attend antenatal clinics? | 1.Yes 2.No | If NO skip to question 303 |
| --- | --- | --- | --- |
| 302a | If yes, where did you attend antenatal clinics? | 1.hospital  2.health center  3. private facility  4. others(specify)____ |  |
| 302b | If yes at ANC, did the provider ever mentioned about you choose your companion in labor? | 1.Yes 2.No |  |
| 303 | Do you have any complications during your labor? | 1. Yes 2. No | **If no to q304** |
| 303a | If yes, type of complications? | 1. Obstetrics 2. Medical |  |
| 304 | Was the pregnancy planned and supported? | 1. Yes 2. No |  |
| 305 | What was the outcome of delivery? | 1. baby alive and well  2. baby was sickly  3. baby dead |  |
| 306 | Do you think that allowing you to choose someone to stay with you would make you eager to deliver health institutions? | 1.yes 2.no |  |
| 307 | Route of delivery | 1. Vaginal 2. C/section |  |

## D. provider’s & facility related information’s

| 401 | Do you think that this facility is comfortable to be accompanied by their choice of companion? | 1.yes 2.no |  |
| --- | --- | --- | --- |
| 402 | Do you think that the care providers in this facility are busy? | 1. Yes 2.no |  |
| 403 | Place of current intra natal and post natal care attaining? | 1. Hospital 2. Health center |  |

## የአማረኛ መጠይቆች

የመረጃ መሰብሰቢያ

በአርባምን ዩኒቨርሲቲ

ሚዋይፈሪት/ት

እኔ ……………………..በዚህ ጥናት እንደ መረጃ ሰብሳቢ ሆኜ የምሰራ ነኝ፡፡

ውድ ተሳታፊችን በአርባ ምን ዩኒቨርሲቲ ከሚዋይፈሪ ት/ት ክፍል ጋር በመተባበር ካሳው በየነ በከሊኒካል ሚዋይፈሪ የማስትሬት ዲግሪ ጥናቱን በከፊል ለማጠናቀቅ ለሚያከናው ነው የምርምር ስራ እንዲጠቅሙበተ ለተለያዩ ክፍሎች የተመደቡ የጥያቄዎችን ዝርዝሮች በዚህ ሰነድ ላይ ያገኛሉ፡፡ከቃለመጠይቁ በኋላ ጭምር ጥያቄዎች ካሉሽ ለመጠየቅ ነጻ ሁኚ፡፡

**የገንዘብ ድጋፍ ያደረገው ድርጅት ስም፡-** አርባምንጭ ዩኒቨርሲቲ

**የጥናቱ አካሄድ**

በዚህ ጥናት እንዲሳተፉ እርስዎ የተመረጡ ሲሆን፡ በዚህ ጥናት እንዲሳተፉ በትህትና ይጠየቃሉ፡፡ ለመሳተፍ ከተስማሙ ስምምነቱን በደንብ መረዳትና እንደተስማሙ በቃል መግለጥይገባዎታል፡፡

**የጥናቱ ዓላማ**

የዚህ ጥናት ዋና አላማ “በምጥ ወቅት አብሮ/ራ ስለሚሆን /ስለምትሆን ግለሰብ የድህረመወሊድ ደንበኞች ያላቸውን ተግባራዊነት እና ተግዳሮቶችን ማጥናትነው

**ጥቅሞች**

በዚህ ጥናት በመሳተፍዎ የሚያገኙት የገንዘብ ጥቅም የለም፡፡በዚህ ጥናት በመሳተፍዎ ባጠቃላይ ምንም አይነት ችግር አይደርስብዎትም ፤ ምናልባት ጊዜዎትን ሊሻማብዎ ይችል ይሆናል፡፡

**ሚስጢር ስለመጠበቅ**

ከዚህ ጥናት የሚገኘው መረጃ ሁለ በሚስጥራዊነት ይጠበቃል፡፡ለዚህ ጥናት የሚሰበሰበው እርስዎን የሚመለከት መረጃ በማህደር የሚቀመጥ ሲሆን ማህደሩም በስም ሳይሆን በተለየ ኮድ ሲቀመጥ ኮዱን ከዋናው ተመራማሪ ውጭ ለማንም አይገለጽም፡፡በጥናቱ ያለመሳተፍ ወይም እራስዎን ለማግለል ወይም በጥናቱ ላለመሳተፍ ከፈልጉ በዚህ ጥናት ያለመሳተፍ ሙሉ መብት አለዎት፡፡በዚህ ጥናት ባለመሳተፎ የሚያጡት አገልግሎት አይኖርም፡፡

**ከአጥኚው ሰው ጋር መገነኛ መንገዶች**

ማንኛውም አይነት ጥያቄ ቢኖርዎት ከዚህ በታች ባለው አድራሻ መጠየቅ ይችላሉ፡፡

- ካሳው በየነ ጌታሁን
- ስልክ ቁጥር፡ 0912136127
- ኢሜል፡ [Kassaw.kb3@gmail.com](mailto:Kassaw.kb3@gmail.com)

**የስምምነት ቅጽ**

የዚህ ጥናት የመሳተፍም ሆነ ያለመሳተፍም መብት እንዳለኝና በጥናቱ በመሳተፌ ምንም አይነት የገንዘብ ጥቅም እንደለለውና በጥናቱ ባለመሳተፌ ምንም አይነት ጉዳት እንደማይደርስብኝ ፣የምሰጠውን መረጃ ለጥናቱ ብቻ እንደሚጠቀሙበት ተነግሮኛል፡፡

እናም በጥናቱ ለመሳተፍ

ሀ/ ተስማምቻለሁ፡፡__________ቀጥል

ለ/ አልስማማም፡፡_____________አቁም

**የጥያቄውመለያቁጥር_________________**

ሀ ፡- **የማህበረሰባዊና ስነ-ህዝብ ጥናት መረጃ**

| ተ.ቁ | ጥያቄዎች | አማራጭ መልሶች | ዝለል |
| --- | --- | --- | --- |
| 101 | እድሜሽ ስንት ነው? | -------------ዓመት |  |
| 102 | የት ነዉ የሚኖሩት ? | 1. ገጠር  2. ከተማ |  |
| 103 | ባሁኑ ወቅት የረስዎ የጋብቻ ሁኔታ | 1. ያገባች  2. ያላገባች  3. የፈታች  4.ጋለሞታ |  |
| 104 | ሃይማኖት | 1) ኦርቶዶክስ  2) ፕሮቴስታንት  3) ሙሰሊም  4) ካቶሊክ  6)ሌሎች (ይግለጹ) ---- |  |
| 105. | ብሔር | 1)ጋሞ  2.ጎፋ  3) ወላይታ  4)አማራ  5) ኦሮሞ  6)ሌሎች(ይግለጹ)_______ |  |
| 106. | የርስዎ የት/ት ሁኔታ? | 1. 1.አልተማረኩም   2. የመጀመሪ ደረጃ   1. 3.የሁለተኛ ደረጃ   4. ከሁለተኛ ደረጃ በላይ |  |
| 107 | ስራወት ምነድን ነዉ? | 1. 1. የቤት እመቤት 2. 2. የመንግስት ሰራተ 3. 3. የግል ስራ/መንግስታዊ ያልሆነ 4. ድርጅት 5. 4. ሌላ ካለ ------------- |  |
| 108 | የባለቤትዎ የት/ት ሁኔታ? | 1.ያልተማረ  2. የመጀመሪ ደረጃ   1. 3.የሁተኛደረጃ 2. 4. ከሁተኛ ደረጃ በላይ |  |
| 109 | የቤተሰበዎ የወር ገቢ ስንት ነው? | ----------ብር |  |

ለ. በምጥ ጊዜ ሊኖርዎት ስለሚች ልረዳት/ድጋፍ ሰጪ ያለውን ተግባራዊነትጥያቄዎች

| ተ.ቁ | ጥያቄወች | የመልስአማራጮች | ይለፉ |
| --- | --- | --- | --- |
| 201 | በዚህ የምጥ ወቅቱ የረዳሽ ሰው ነበር? | 1. አዎ አለ  2. የለም | የለም ከሆነ ወደ 201ለ እለፍ |
| 201ሀ | በወቅቱ የረዳሽ ሰው ካለ ማን ነበር? | 1) ባለቤቴ  2) እህቴ/አይቴ ወንድሜ  3)እናቴ አማቴ  4) ጓደኛዬ ጎረቢቴ  5) ሌሎች ( ይግለጹ)_____ |  |
| 201ለ | የረዳሽ ሰው ከሌለ ለምን? | 1) ዕኔ ስላልፈለኩ  2) የጤና ተቋሙ ስለማይፈቅድ  3) የጤና ባለሙያው ባለመፍቀዱ  4) የሚረዳኝ ስለሌለ  5)ሌሎች ( ይግለጹ)_____ |  |
| 202 | በዚህ የወሊድ ወቅቱ የረዳሽ ሰው ነበር? | 1. አዎ አለ 2. የለም |  |
| 202ሀ | በወቅቱ የረዳሽ ሰው ካለ ማን ነበር? | 1) ባለቤቴ  2) እህቴ  3)እናቴ  4) ጓደኛዬ  5) ሌሎች ( ይግለጹ)_____ |  |
| 202ለ | የረዳሽ ሰው ከሌለ ለምን? | 1) ዕኔ ስላልፈለኩ  2) የጤና ተቋሙ ስለማይፈቅድ  3) የጤና ባለሙያው ባለመፍቀዱ  4) የሚረዳኝ ስለሌለ  5)በኦፕሬሽን ሰስለወለድኩ |  |

ሐ. በምጥ ወቅት አብሯት ስለሚሆንና እና ስሚረዳት ሰው ያላት እውቀት እና ፍላጎት

| 204 | በአንድ የጤና ተቋም ውስጥ በምጥ ጊዜ በቤተሰብ አባላት ስለሚደረግ እገዛ እውቅ አለሽ | 1) አዎ  2) አላውቅም | አላውቅም ከሆነ መልሱ ወደጥያቄ 205 እለፊ |
| --- | --- | --- | --- |
| 204ሀ | አዎ ከሆነ ምን ማለት ነው | 1.በምት ጊዜ የሚደረግ እገዛ  2.በእርግዝና ጊዜ የሚደረግ እገዛ  3. ከወሊድ በሁላ የሚደረግ እገዛ  4. ሌላ ካለ------ |  |
| 205 | በምጥ ጊዜ በቤተሰብ አባላት እገዛ መደረጉ ውጤቱ አወንታዊ ይሆናል ብለሽ ታምኛለሽ | 1. አዎ አምናለሁ 2. አይ አላምንም |  |
| 206 | በምጥ ላይ ያለች ሴት በአንድ የጤና ተቋም ውስጥ ልጇ እስክትገላገል ድረስ አብሯት እንዲቆይ እና እንዲደግፋት የምትፈልገውን ሰው የመምረጥ መብት እንዳላት ታውቂያለሽ? | 1) አዎ  2) አላውቅም | አላውቅም ከሆነ መልሱወደ 207 እለፊ |
| 206ሀ | አዎ ከሆነ እንዴት ልታውቂ ቻልሽ? ውይም ከየት ነው መረጃውን ያገነኙት? | 1)ከሰዎች ሰምቼ  2) ከዚህ በፊት ተሞክሮ ስላለኝ  3) ከጤና አገልግሎት ሰጪዎች ሰምቸ  4) አንብቤ  5)ሌሎች ( ይግለጹ)___ |  |
| 207 | በምጥ እና በመውለድ ወቅትየሚረዳሽ አንድ ሰው አብሮ ስለመሆኑየምትይው ነገር ምንድ ነው? | 1) በጣም ጥሩ አሰራርነው  2) ጥሩ አሰራር አይደለም |  |
| 208 | በምጥ ጊዜ አብሮሽ የሚሆን ሰዉ እንዲደርግልሽ የምትፈልጊዉ ነገር አለ | 1.አዎ አለ  2.የለም |  |
| 208ሀ | በምጥ ወቅት አብሮሽ ያለ ግለሰብ ምን እንዲያደርጉልሽ ትጠብቂያለ? ( ከአንድ በላይ መልስ መምረጥ ይቻላል) | 1)እንዲያበረታታኝ  2)ጀርባሽን እንዲያሽልኝ  3)እንዲጸልይልሽ  4) ወጭ የምትፈልጊውን ነገር እንዲያቀርብልሽ  5)ሌላ ( ይገለጽ)_____ |  |
| 209 | በምጥ ወቅት የሚረዳሽ ሰው አብሮሽ ቢኖርያለውን ጥቅም ታውቂለሽ? | 1. 1.አወአውቃለሁ 2. 2.አላውቅም | አላውቅም ከሆነ ወደ2010 እለፉ |
| 209ሀ | በምጥ ወቅት የሚረዳሽ ሰውቢኖር ሊያስገኘው ስለሚችለው ጥቅም የምታውቂው ነገር አለ?  ( ከአንድበላይመልስመምረጥይችላሉ) | 1)በምጥ ጊዜ ህመም ይቀንሳል | |
|  |  |  |  |
|  |  | 2)በቀዶ ጥገና የመውለድ አስፈላጊነት ይቀንሳል | |
|  |  |  |  |
|  |  | 3) በብልት ( ቫጃይና) የመውለድ እድል እንዲጨምር ያደርጋል | |
|  |  |  |  |
|  |  | 4)በሴቷ ውስጥ ያለጭንቀትና ፍርሃት እንዲቀንስ ይረዳል | |
|  |  |  |  |
|  |  | 5)ሴቷን ደስ ያሰኛታል | |
|  |  |  |  |
|  |  | 6) የምጥ ጊዜ እንዲቀንስ ያደርጋ | |
|  |  |  |  |
|  |  | 7)የጨቅላውን በህይወት የመቆየት እድል የተሻለ ያደርጋል | |
|  |  |  |  |
|  |  | 8)እናቶች የሚሰማቸውን የብቸኝነት ስሜት ይቀንሳል | |
|  |  |  |  |
|  |  | 9) ሴቷ በጤና አገልግሎት ሰጪዎች  የመጠቃትና ክብሯን የማጣት እድልን ይቀንሳል | |
|  |  |  |  |
|  |  | 10. ሌላም ካለ ይግለጹ------ | |
|  | በምጥ ወቅት ሰው አብሯት እንዲሆንና እና እንዲረዳት ያላት ፍላጎት |  | |
| 210 | በምጥ ጊዜ የሚረዳሽ ሰው አብሮሽ እንዲሆን ፍላጎቱ አለሽ | 1. አዎ 2. አልፈልግም | |
| 210ሀ | ፍላጎት የለኝም ከሆነ ለምን? | 1.አብሮኝ ላለ ሰው ተጋላጭ ላለመሆን  2.ብቻየን መሆን ስለምፈልግ  3. ሌላ ምክኒያት--------------- | |

መ. ከቀደሞው እና ከአሁኑ ከእርግዝና እና ምጥ ጋር የተያያዙ ጥያቄዎች

| ተ.ቁ | ጥያቄዎች | የመልስአማራጮች | ዝለል |
| --- | --- | --- | --- |
| 301 | ከዚህ በፊት ስንት ልጆች ወልደሻ? | በቁጥር_________ | መልሱ የለም ከሆነ ወደ ክፍል መ እለፉ |
| 301ሀ | በዚያን ጊዜ ልጅሽን የወለድሽው የት ነበር? | 1) በቤቴ  2) ጤናጣቢያ  3) የመንግስትሆፒታል  4) የግልሆ/ልወይምክሊኒክ  5)ሌሎች (ይገለጽ) | መልሱ 1 ካልሆነ ወደ 302 ይለፉ |
| 30ለ | በቤት ውስጥ ከወለድሽ ለምን?  ( ተገቢ ከሆነ ከ1 በላይ መልስን መስጠት ትችያለሽ) | 1) ባህላዊ አዋላጆችን ስለምመርጥ  2) የተለመደ ስለሆነ  3) እርግዝናዬ የጤና ችግር ስለሌለ  4) በጤና አገልግሎት ሰጪ እንዳልንገላታና ክብሬን እንዳላጣ  5) በምጥ ወቅት ብቻዬን መሆን ስለምፈራ  6) ያማጥሁት ለአጭር ጊዜ በመሆኑ  7) የትራንስፖርት ችግር ስላለ  8) ሌሎች (ይግለጹ)_ |  |

| 302 | የቅድመ ወሊድ ክትትል ነበርሽ? | | 1) አዎ  2) አልተከታተልሁም | አልተከታተልምከሆነ ወደጥያቄ 303 ይለፉ |
| --- | --- | --- | --- | --- |
| 302ሀ | ዐዎ ካሉ የቅድመ ወሊድ ክትትል የተከታተልሽው የት ነበር? |  | 1) የጤና ጣቢያ  2) የመንግስትሆስፒታል  3) የግል ተቋም  4) ሌሎች ( ይገለጽ)____ | |
| 302ለ | ዐዎ ካሉ በዚህ ተቋም ውስጥ በምጥ ወቅት አብሮሽ የሚሆን ሰው መምረጥ እንደምትችይ ገለጻ ተደርጎልሽ ያውቃል? | | 1) ገለጻ ተደርጎልኛል  2) ገለጻ አልተደረገልኝም |  |
| 303 | በምጥሽ ወቅት የተለየ የጤና ችግር ገጥሞሽ ነበር | | 1.አዎ 2. አልነበረም | 2 ከሆነ ወደ 304 |
| 303ሀ | አዎ ከኆነ የገጠመሽ ችግር ምን ነበር | | 1 .ከርግዝና ጋር ተያያዝ  2. ሜዲካለ ችግር |  |
| 304 | ይህ ዕርግዝና የታቀደና የተፈለገ ነው | | 1.አዎ 2. ዐይደለም |  |
| 305 | የወለሽው ልጅ የጤና ሁኔታ እንዴት ነበር? | | 1.ጤነኛ ነበር  2. የታመመ ነበር  3. ህይወቱ ያለፈ ነበር |  |
| 306 | በዚህ ተቋም ውስጥ በምጥወቅት አብሮሽ እንዲቆይ የምትፈልጊውን ሰው እንድትመርጪ ቢፈቀድልሽ ይህ በጤና ተቋሙ ውስጥ ለመውለድ ፍላጎት ሊያሳድርብሽ ይችላል? | | 1) አዎ  2) የለም |  |
| 307 | የወሊድ አይነት | | 1.በብልቴ 2. በኦፕሬሽን |  |

መ. ከጤና ተቑማትና ከባለሙያዎች ጋር የተያያዙ መረጃዎች

| **401** | በዚህ ተቁም በምጥ ወቅት አብሮሽ ለሚሆን ሰው ሁኔታወቹ መቹ ይመስሉሻል? | 1. አዎ ነው  2. አይደለም |  |
| --- | --- | --- | --- |
| **402** | በዚህ ተቋም ውስጥ የሚሰሩ ባለሙያዎች ስራ ይበዛባቸዋል ብለሽ ታሽቢያለሽ ? | 1. አዎ ይነዛባቸዋል  2. አይበዛባቸዉም |  |
| **403** | አሁን የድህረ ወሊድ አገልግሎት ያገኘሽበት ተቃም | 1.ሆስፒታል 2.ጤና ጣቢያ |  |

**ስለተሳትፎዎ አመሰግናለሁ**
