# Supplementary material for: Utilization of companionship during delivery and associated factors among women who gave birth at Arba Minch town public health facilities, southern Ethiopia
Source: PLoS One. 2020 Oct 2;15(10):e0240239. doi: 10.1371/journal.pone.0240239 (PMC7531811; doi:10.1371/journal.pone.0240239)
Supplement: Attachment — Submitted filename: PONE-D-20-18273_reviewer-1.pdf [file pone.0240239.s003.pdf]

PLOS ONE

Utilization of Companionship during delivery and associated factors among women who gave birth at Arbaminch town public health facilities, southern Ethiopia

--Manuscript Draft--

**Manuscript Number:**	PONE-D-20-18273
**Article Type:**	Research Article
**Full Title:**	Utilization of Companionship during delivery and associated factors among women who gave birth at Arbaminch town public health facilities, southern Ethiopia
**Short Title:**	Utilization of companion during child birth
**Corresponding Author:**	Biresaw Wassihun Alemu, Msc<br>Arba Minch University<br>Arba minch, SNNPR ETHIOPIA
**Keywords:**	companionship; Arbaminch town; delivery; and utilization
**Abstract:**	Background<br><br>Companionship during delivery is an important feature of compassionate and respectful maternity care. It has a positive impact on delivery and birth outcomes. In low resource countries like Ethiopia lack of companionship discourages women from accessing facility-based delivery care. However, it doesn’t get appropriate attention. Therefore this study aimed to assess the utilization of companionship during delivery and associated factors.<br><br>Methods<br><br>Health facility-based cross-sectional study design was done from October to November 2019. Interviewer administered questionnaires were used to collect the data from 418 study participants. The data were entered with Epi data version 4.4 and exported to Statistical Package for Social Sciences (SPSS) version 25.0 for analysis. Binary logistic regression was done. Statistical significance was declared at P- values < 0.05 with a 95% confidence level .<br><br>Results<br><br>The finding of the study showed that only 13.8% of mothers utilize companionship during delivery. Variables such as having a desire to have companionship during delivery in the health facilities (AOR= 5.17, CI 95% 2.63, 10.16), having complication during the labor and delivery (AOR= 3.48, CI 95%, 1.81, 6.70) and being primipara (AOR= 2.05, CI 95% 1.09, 3.87) were the independent factors associated with companionship utilization.<br><br>Conclusions<br><br>The finding of the study showed that the utilization of companionship during delivery was low. Permitting women to have a companion of choice during labor and childbirth can be a cost-effective intervention to improve the quality of maternity care, facing complications during delivery, having a desire to have companionship during delivery and primiparous women were more likely to utilize companionship. To improve this low utilization of companionship institutions and care providers should provide information about companionship during antenatal care attendance. Besides, there is a need for clear guidelines to govern the practice of companions
**Order of Authors:**	Kassaw Beyene
	Biresaw Wassihun Alemu, Msc
**Additional Information:**	
**Question**	**Response**

Powered by Editorial Manager® and ProduXion Manager® from Aries Systems Corporation

**Financial Disclosure**<br><br>Enter a financial disclosure statement that describes the sources of funding for the work included in this submission. Review the submission guidelines for detailed requirements. View published research articles from *PLOS ONE* for specific examples.<br><br>This statement is required for submission and **will appear in the published article** if the submission is accepted. Please make sure it is accurate.<br><br>**Unfunded studies**<br>Enter: *The author(s) received no specific funding for this work.*<br><br>**Funded studies**<br>Enter a statement with the following details:<br>• Initials of the authors who received each award<br>• Grant numbers awarded to each author<br>• The full name of each funder<br>• URL of each funder website<br>• Did the sponsors or funders play any role in the study design, data collection and analysis, decision to publish, or preparation of the manuscript?<br>• **NO** - Include this sentence at the end of your statement: *The funders had no role in study design, data collection and analysis, decision to publish, or preparation of the manuscript.*<br>• **YES** - Specify the role(s) played.<br><br>* typeset	no
**Competing Interests**<br><br>Use the instructions below to enter a competing interest statement for this submission. On behalf of all authors, disclose any competing interests that could be perceived to bias this work—acknowledging all financial support and any other relevant financial or non-financial competing interests.<br><br>This statement **will appear in the published article** if the submission is accepted. Please make sure it is	no authors have competing interests

accurate. View published research articles from *PLOS ONE* for specific examples.<br><br>**NO authors have competing interests**<br><br>Enter: *The authors have declared that no competing interests exist.*<br><br>**Authors with competing interests**<br><br>Enter competing interest details beginning with this statement:<br><br>*I have read the journal's policy and the authors of this manuscript have the following competing interests: [insert competing interests here]*<br><br>* typeset	
**Ethics Statement**<br><br>Enter an ethics statement for this submission. This statement is required if the study involved:<br><br>• Human participants<br>• Human specimens or tissue<br>• Vertebrate animals or cephalopods<br>• Vertebrate embryos or tissues<br>• Field research<br><br>Write "N/A" if the submission does not require an ethics statement.<br><br>General guidance is provided below. Consult the submission guidelines for detailed instructions. **Make sure that all information entered here is included in the Methods section of the manuscript.**	not applicable

**Format for specific study types**<br><br>**Human Subject Research (involving human participants and/or tissue)**<br>• Give the name of the institutional review board or ethics committee that approved the study<br>• Include the approval number and/or a statement indicating approval of this research<br>• Indicate the form of consent obtained (written/oral) or the reason that consent was not obtained (e.g. the data were analyzed anonymously)<br><br>**Animal Research (involving vertebrate animals, embryos or tissues)**<br>• Provide the name of the Institutional Animal Care and Use Committee (IACUC) or other relevant ethics board that reviewed the study protocol, and indicate whether they approved this research or granted a formal waiver of ethical approval<br>• Include an approval number if one was obtained<br>• If the study involved *non-human primates*, add *additional details* about animal welfare and steps taken to ameliorate suffering<br>• If anesthesia, euthanasia, or any kind of animal sacrifice is part of the study, include briefly which substances and/or methods were applied<br><br>**Field Research**<br><br>Include the following details if this study involves the collection of plant, animal, or other materials from a natural setting:<br>• Field permit number<br>• Name of the institution or relevant body that granted permission	
**Data Availability**<br><br>Authors are required to make all data underlying the findings described fully available, without restriction, and from the time of publication. PLOS allows rare exceptions to address legal and ethical concerns. See the PLOS Data Policy and FAQ for detailed information.	Yes - all data are fully available without restriction

A Data Availability Statement describing where the data can be found is required at submission. Your answers to this question constitute the Data Availability Statement and **will be published in the article**, if accepted.<br><br>**Important:** Stating 'data available on request from the author' is not sufficient. If your data are only available upon request, select 'No' for the first question and explain your exceptional situation in the text box.<br><br>Do the authors confirm that all data underlying the findings described in their manuscript are fully available without restriction?	
**Describe where the data may be found in full sentences. If you are copying our sample text, replace any instances of XXX with the appropriate details.**<br><br>• If the data are **held or will be held in a public repository**, include URLs, accession numbers or DOIs. If this information will only be available after acceptance, indicate this by ticking the box below. For example: *All XXX files are available from the XXX database (accession number(s) XXX, XXX.*).<br>• If the data are all contained **within the manuscript and/or Supporting Information files**, enter the following: *All relevant data are within the manuscript and its Supporting Information files.*<br>• If neither of these applies but you are able to provide **details of access elsewhere**, with or without limitations, please do so. For example:<br><br>*Data cannot be shared publicly because of [XXX]. Data are available from the XXX Institutional Data Access / Ethics Committee (contact via XXX) for researchers who meet the criteria for access to confidential data.*<br><br>*The data underlying the results presented in the study are available from (include the name of the third party*	data is available on Author

and contact information or URL).<br>• This text is appropriate if the data are owned by a third party and authors do not have permission to share the data.<br><br>* typeset	
Additional data availability information:	

Powered by Editorial Manager® and ProduXion Manager® from Aries Systems Corporation

Manuscript

Click here to download Manuscript CDD Munscript kassiaw.docx

Utilization of Companionship during delivery and associated factors among women who gave birth at Arbaminch town public health facilities, southern Ethiopia

Kassaw Beyene[1] Biresaw Wassihun*[¶1]

[1]Arba Minch University, College of Medicine and Health Science, Department of Midwifery, Arba Minch, Ethiopia

* Corresponding author

Biresaw Wassihun: Email: bireswas@gmail.com

[¶]These author contributed equally to this work

ABSTRACT

Background: Companionship during delivery is an important feature of compassionate and respectful maternity care. It has a positive impact on delivery and birth outcomes. In low resource countries like Ethiopia lack of companionship discourages women from accessing facility-based delivery care. However, it doesn't get appropriate attention. Therefore this study aimed to assess the utilization of companionship during delivery and associated factors.

Methods: Health facility-based cross-sectional study design was done from October to November 2019. Interviewer administered questionnaires were used to collect the data from 418 study participants. The data were entered with Epi data version 4.4 and exported to Statistical Package for Social Sciences (SPSS) version 25.0 for analysis. Binary logistic regression was done. Statistical significance was declared at P- values < 0.05 with a 95% confidence level .

Results: The finding of the study showed that only 13.8% of mothers utilize companionship during delivery. Variables such as having a desire to have companionship during delivery in the health facilities (AOR= 5.17, CI 95% 2.63, 10.16), having complication during the labor and delivery (AOR= 3.48, CI 95%, 1.81, 6.70) and being primipara (AOR= 2.05, CI 95% 1.09, 3.87) were the independent factors associated with companionship utilization.

Conclusions: The finding of the study showed that the utilization of companionship during delivery was low. Permitting women to have a companion of choice during labor and childbirth can be a cost-effective intervention to improve the quality of maternity care, facing complications during delivery, having a desire to have companionship during delivery and primiparous women were more likely to utilize companionship. To improve this low utilization of companionship institutions and care providers should provide information about companionship during antenatal care attendance. Besides, there is a need for clear guidelines to govern the practice of companions

Keywords: companionship, Arbaminch town, delivery, and utilization.

Background

Providing continuous emotional and physical support during delivery is one of the important measures of quality of care in the health facility because it is one of the potential ways to advance the quality of maternity care (1).

Labor is considered as periods of developing extreme anxiety, fear, emotional disturbance, and stress in women's life. So providing continuous emotional support is vital for achieving positive outcomes by enhancing the physiological process of labor (2). Having a labor companion is likely to cope with labor induced stress and adapt to a strange environment when someone she knows is continuously assisted her, encouraging, and appreciating her efforts during delivery(3,4).

World health organization(WHO) recommends that facilitating and ensuring clear and respectful communication between health-care providers and the woman in labor, and providing continuous emotional support is advocated for all women. However, in the actual clinical setting, it not well-practiced(5-6). Permitting and supporting the presence of a woman's companion of choice during labor and childbirth is vital to reduce mistreatment or abuse in a health facility (6) .beside it will increase women's satisfaction with their experience of childbirth and reduce the risk of medical interventions, like emergency cesarean section (7).

Companionship during delivery is one of the core components of respectful maternity care (RMC), but it is a neglected area. (8,9)

A previous study showed that the low practice of labor companions was associated with the absence of guidelines, lack of infrastructure to protect privacy, overcrowding of ward and poor knowledge and negative attitudes of health-care providers (10,11).

Now a day, labor companion is increasingly being included in the maternal health guidelines of many countries, however, little is known about the extent to which labor companionship is practiced especially, in most low-income countries (11, 12).

In Ethiopia, companionship during delivery is not well studied. Therefore, this study is aimed to assess the utilization of companionship during childbirth and associated factors among women who give birth at Arba Minch town public health facilities, South Ethiopia.

Methods

Study setting and Study period

The study was conducted in Arbaminch town public health facilities from October to November 2019. Arbaminch town is the administrative city of the Gamo zone, southern Ethiopia, which is 454km south of Addis Ababa (the capital city of Ethiopia) and about 280 Km from Hawassa (the capital of SNNP). The town is subdivided into 4-sub city and 11 kebeles (the smallest administrative structure in Ethiopia). The town has a total area of 5556 hectares and a total population of 112,724 among those (50.2%) of them were females. The number of health institutions in Arba Minch town is 1 governmental general hospital, 2 health centers, 33 private clinics, 12 drug store, and 2 community pharmacy

Study design

A health facility-based cross-sectional study design was carried out.

Populations

Source population

All women who gave birth at Arba Minch town public health facilities.

Study population

All women who gave birth in Arba Minch town public health facilities during the study period.

Inclusion and Exclusion criteria

Inclusion criteria

All women who were laboring a[illegible]ave birth at Arba Minch town public health facilities.

Exclusion criter[illegible]

Those women who are seriously ill and unable to communicate.

Sample size determination

The sample size was calculated using a single population proportion formula by considering the following assumptions: 95% confidence level, the margin of error (0.05), p= 44.7%(13). The required sample size after adding 10% non-response rate was 418

Sampling techniques & procedure

There is one public hospital (Arbaminch general hospital) and two public health centers (Sikela and Shecha health centers) in Arbaminch town and all were included in the study. The allocation of the sample to health facilities was made proportionally based on the number of women who give birth at each facility in the two months preceding the data collection period.

Individual study subject at each health facility was selected by systematic random sampling during the data collection period until the required sample size at each health facility was obtained

The sampling interval k=2 was calculated by dividing the source population to the total sample size and this interval was used in all health facilities to select study subjects.

Therefore, the first women from each health institution were selected by lottery method. Then every other woman from each health institution was interviewed.

Operational definitions

Labor companionship: support provided to laboring women at all moments of the labor process. It may be provided by a partner, family member, or social network(14).

Utilization of companionship: having a support person of laboring women to provide support and stay with her during labor in the health facilities.

Data collection tools

A structured questionnaire was used to collect data from the postnatal women in the labor ward of each health facility. Trained interviewers were administered the questionnaire after obtaining written consent from each postnatal woman. The questionnaire contained information about socio-demographic characteristics, obstetric characteristics, knowledge about a companion in labor, desire to have a companion in labor, facilities, and health care provider's characteristics and utilization of companionship during labor. The questionnaire was developed in English language and then translated into Amharic. The Amharic version of the questionnaire was translated back into the English language to confirm the correct and precise interpretation of the questionnaire. Six diploma midwives data collectors and three BSc midwives supervisors were recruited for data collection.

Data quality control

The pretest was done on 5 % of the sample size (by 21 questionnaires) to ensure its consistency and validity then correction was made accordingly before the actual data collection. One day training was given for data collectors and supervisors about the methodology and questionnaires by the principal investigator. During the data collection period, Study participants were informed about the purpose of data collection and the importance of the study to generate quality data. The collected data were checked for completeness and consistencies by trained supervisors and

investigators daily and immediate action was taken accordingly. After data collection, the collected data were rechecked for completeness and consistencies by the investigator.

Data processing and analysis

The collected data was checked by the principal investigator, and then data were coded, entered, and cleaned using Epi Data version 4.4 software and finally exported into SPSS version 25.0 software for analysis. Descriptive statistics of different variables were determined and the result was presented in tables, charts, and graphs using summary measures such as percentages mean and median. Binary logistic regression was used for the analysis of the outcome variable.

A Hosmer-Lemeshow test was done to test for model fitness. Fist bivariate analysis was carried out to identify the factors associated with utilization of labor companionship than multivariable analysis was done by considering a p-value of < 0.25, biological plausibility and consistency in the previous studies to see the independent effect of each variable on the outcome variable. The multi co-linearity test was carried out to see the correlation between independent variables.

Finally, the result of bivariate and multivariable logistic regression analysis was presented in a crude and adjusted odds ratio with 95% confidence intervals. $P< 0.05$ was considered statistically significant.

Ethical consideration

Ethical clearance was obtained from the institutional Research Ethics review board of the colleg of medicine and health science, Arba Minch University. Permission was obtained from the managers of each health facility. All participants were informed about the objectives of the study and that their participation was voluntary. It was also clearly stated to the participants that the information they provided was for research purposes and strictly confidential. Data were collected before discharge to home after she was stable and comfortable.

RESULTS

Socio-demographic characteristics of respondents

Four-hundred seven women participated in the study with a 97.3% response rate. The mean age of the study participants was 26 years (SD± 4.86 years) and 181 (44.5%) women were between the age group of 25 – 34 years. 281 (69%) of respondents were urban residents and 386 (94.8%) of the study participants were married. Among the total respondents, 146(35.9%) of women had primary educational level and One hundred ninety-seven (48.4%) were Orthodox Christians.

Half of the respondents were from Gamo ethnic group (51.4%) followed by Gofa ethnic group 68(16.7%) (**Table 1**).

Table 1: Socio-demographic characteristics of the study participants, Arba Minch town, south Ethiopia, 2019, (n=407).

Variables	Frequency	Percentage
Age		
<25	171	42
25-34	181	44.5
≥35	55	13.5
Residency		
Rural	126	31
Urban	281	69
Marital status		
Married	386	94.8
Single	11	2.7
Divorced	8	2
Widowed	2	0.5
Religion		
Orthodox	197	48.4
Protestant	162	39.8
Muslim	31	7.6
Catholic	14	3.4
Others*	3	0.7
Ethnicity		
Gamo	209	51.4
Gofa	68	16.7
Welayta	25	6.1
Amhara	33	8.1
Oromo	21	5.2
Others**	51	12.5
Educational level		
No formal education	87	21.4
primary	146	35.9
Secondary	105	25.8
Above secondary	69	17.0
Occupation		
Housewife	212	52.1
Government employee	69	17.0

NGO/private	99	24.3
Others	27	6.6
Husband educational level		
No formal education	69	17.0
Primary	102	25.1
Secondary	133	32.7
Above secondary	103	25.3

Key=*traditional, Jehovah witness, **Konso, Derashe, Gurage, Amaro, ^ Students, Daily labor

Obstetrics characteristics of the respondents

Two hundred thirty (56.5%) of the study participants were multiparous and 54(29.3%) had labor companionship during previous institution delivery. Almost all 363 (89.2%) women had antenatal follow up during current pregnancy and only 45 (12.4%) of women had got information from health care providers about labor companionship during antenatal care attendance.

Majority 346(85%) of respondents perceived that allowing laboring women to have a companion during childbirth. Of the total respondent, 387(95.1%) of them had planned pregnancy (**Table 2**).

Table 2: Obstetrics characteristics of women who give birth in Arbaminch town public health facilities southern Ethiopia, 2019

Variables	Frequency	Percentage
Parity		
Primipara	177	43.5
Multipara	230	56.5
Have companion during the last delivery(n=230)		
Yes	54	29.3
No	130	70.7
Place of last delivery(n=230)		
Home	48	20.9
Health institutions	182	79.1
Reason to deliver at home* (n=48)		
To be attended by TBAs	32	66.7
Free of abuse & disrespect	16	33.3
Did you attend ANC in your current pregnancy (n=407)		
Yes	363	89.2
No	44	10.8
Where do you attend (n=363)		

At hospital	108	29.8
At the health center	235	64.7
At the private health facility	20	5.5
Did the provider inform you about companion(n=363)		
Yes	45	12.4
No	318	87.6
Status of Pregnancy		
Planned	387	95.1
Unplanned	20	4.9
Do you have a facing complication during pregnancy and labor (n=407)		
Yes	87	21.4
No	320	78.6
Type of complications (n=87)		
Obstetrics	62	71.3
Medical	25	28.7

*=variables not sum up out of 100%

Benefits of companionship during delivery

Majority of respondent 77.2% mention that having companionship during delivery can reduce loneliness followed by reducing labor pain management **(figure 1**)

Utilization of companionship during delivery

Of the total respondents, 56 (13.8%) of laboring mothers utilize companionship during delivery, and 351(86.2%) not utilize companionship. The main reason mentioned for not to utilize companionship during delivery was provider denial 47.9% followed by an institution not allowed 21.1% (**Figure 2.)**

Factors associated with having a companion during delivery

To determine the association between utilization of companionship during delivery in the health facilities with different factors, the following dependent variables were checked against outcome variables. On bivariate analysis, women's occupation, family monthly income, complication during labor and delivery, parity of woman, Comfortability of facilities to be accompanied and knowledge had significantly associated with utilization of companion during delivery in the health facilities.

After controlling the effects of confounder on multivariable analysis, having a desire to be accompanied, and complication during labor and delivery and parity have a statistically

significant association with utilization of companionship during delivery**.** Respondents who had a desire to be accompanied during labor and delivery were 5 times more likely to accompanied by their companion than others (AOR=5.17 (2.63, 10.16). Those respondents who have had complications in the current pregnancy and labor were 3,48 times more likely to utilize their companionship than others (AOR=3.48 (181, 6.70). Besides, those respondents who gave birth for the first time (primipara) were 2.05 times more likely to have been accompanied by their companion than multiparous women (AOR= 2.05, 1.09, 3.87) (**Table .3)**

Table 3. Bivariate and multivariable analysis of factors associated with utilization of companion during delivery, Arbaminch Ethiopia, Feb 2019 (n=407)

Variables	Having companion during delivery		Odds Ratio with 95% CI	
	Yes	No	COR	AOR
**Women occupation**				
Unemployed	25	187	1	1
Employed	31	164	0.70(0.40, ,1.24)	1.48(0.77, 2.78)
**Monthly family income**				
≥3000 ETB	29	211	0.71(0.40, 1.25)	0.52(0.27, 1.02)
<3000 ETB	27	140	1	
**Desire to have a companion during delivery**				
Yes	42	135	4.80(2.52 , 9.12)	5.17(2.63, 10.16)*
No	14	216	1	
**Complications during pregnancy & labor**				
Yes	24	63	3.42(1.89, 6.21)	3.48(181,6.70)**
No	32	288	1	1
**Parity**				
Primipara	35	142	2.45(1.37, 4.38)	2.05(1.09, 3.87)*
Multiparous	21	209	1	1
**the facility was comfortable to**				

**be accompanied**				
Yes	15	70	1.46(0.76, 2.80)	1.50(0.73, 3.08)
No	41	281	1	1
**Knowledge**				
Good	6	46	0.79(0.32, 1.96)	0.84(0.31, 2.26)
Poor	50	305	1	1

*=p-value <0.05, **=p-value≤ 0.01, CI = Confidence Interval, COR = Crude Odds Ratio, AOR=Adjusted Odds Ratio.

DISCUSSIONS

Permitting companionship in the health facilities during labor is one of the factors speculated to influence women's decisions to seek skilled birth attendants (14).

The overall utilization of companionship during delivery in the health facilities among study participants was found to be 13.8%. The finding of this study is to lower the study finding done in Brazil by 38.1%, Kenya 67%, and South Africa (15,16,17).

This variation could be due to the cultural difference in labor companion and policy that enforce health care providers to allow labor companion and also study design difference.

The finding of this study showed that being primiparous (delivered for the first time) were two times more likely to be accompanied by their labor companion during childbirth in the health facilities than those women who were multiparous. This finding is similar to the study done in Brazil, which revealed that being primiparous (delivered for the first time) were more likely to be accompanied by their labor companion during childbirth in the health facilities than those women who were multiparous (15). This might be due to the fear of childbirth because most of the time primiparous women may face the fear of childbirth and they will be more likely accompanied by their companion and this fear of childbirth can harm a woman's psychological wellbeing and associated with adverse obstetric outcomes and postpartum mental health difficulties. Women who had obstetrics or medical complications during labor and delivery were 3.48 times more likely to be utilized, labor companion, compared to those women who had never been experiencing any complications during labor and delivery. This finding is supported by a study conducted in Tanzania; parturient women who develop complications during childbirth had significantly greater odds of having companionship during delivery than women who had

normal labor and delivery (13). But in contrast, a study in Kenya(16) showed that women who had experienced complications at labor are 66% less likely to have companionship while giving birth in the health facilities. This difference may be encountered due to women with labor and delivery complication needs strict follow up by health care provider alone, to provide appropriate management without intervention, and to avoid additional stress by her family members.

Conclusion

The finding of this study showed that the utilization of companionship during delivery was low as compared to the previous study. Some of the factors associated with utilization of companionship during delivery was having a desire to companionship, being primiparous, and having facing complication during pregnancy and delivery. Allowing women to have a companion of choice during delivery can be a low-cost and effective intervention to improve the quality of maternity care. To reduce maternal mortality and morbidity provision of and access to a health facility is not enough but improving quality of care is one of the integral components of maternal and child health

List of abbreviations

AGH: Arbaminch General Hospital, ANC: Antenatal Care, CDD: Companionship during Delivery, RMC: Respectful Maternity Care, SSA: Sub Saharan Africa, WHO: World Health Organization

Consent for publication

Not applicable

Competing interests

The authors declare that they have no competing interests.

Funding

Arba Minch University as a requirement for postgraduate studies supports this research financially. The university has no role in the design of the study, collection, analysis, and interpretation of the data and in writing the manuscript.

Acknowledgment

The authors thank all the study participants and Arba Minch University for financial support.

Author's contribution

Conceptualization: Biresaw Wassihun and Kassaw Beyene

Formal analysis: Biresaw Wassihun and Kassaw Beyene

Funding acquisition: Biresaw Wassihun and Kassaw Beyene

Investigation: Biresaw Wassihun and Kassaw Beyene

Methodology Biresaw Wassihun and Kassaw Beyene

Project administration: Biresaw Wassihun and Kassaw Beyene

Resources: Kassaw Beyene and Biresaw Wassihun

Supervision: Biresaw Wassihun and Kassaw Beyene

Validation: Biresaw Wassihun and Kassaw Beyene

Visualization: Kassaw Beyene and Biresaw Wassihun

Writing-original draft: Biresaw Wassihun and Kassaw Beyene

Writing – review & editing: Kassaw Beyene and Biresaw Wassihun

References

1. Hodnett E, Gates S, Hofmeyr G, Sakala C. Continuous support for women during childbirth. Cochrane database of systematic reviews. 2012(10).
2. Banda G. et al. Acceptability and experience of supportive companionship during childbirth in Malawi. BJOG. 2010;117(8):937–45.
3. Sewit G.et al, Knowledge, Attitude, and Practice of Health Professionals Towards Labor Companion in Health Institutions in Addis Ababa International Journal of Women's Health Care. 2018;3(2).
4. Hodnett E, Gates S, Hofmeyr G, Sakala C, Weston J. Continuous support for women during childbirth. Cochrane Database Syst Rev. 2011;2(2).
5. Bohren M, et al. Mistreatment of women during childbirth in Abuja, Nigeria: a qualitative study on perceptions and experiences of women and healthcare providers. Reproductive health. 2017;14(1):9.
6. WHO. Standards for improving the quality of maternal and newborn care in health facilities. WHO. 2017(12).
7. Gülmezoglu A, et al. Interventions to Reduce Maternal and Newborn Morbidity and Mortality.The International Bank for Reconstruction and Development / The World Bank; 2016 Apr 5 Chapter 7 Available from 2016(2).

8. Kaba M, Bulto T, Tafesse Z, Lingerh W, Ali I. Sociocultural determinants of home delivery in Ethiopia: a qualitative study. International journal of women's health. 2016;8:93.
9. Raven J, Tao F, Kun H, Tolhurst R. The quality of childbirth care in China: women's voices: a qualitative study. BMC Pregnancy and Childbirth 2015;1(14):113.
10. Kabakian- Khasholian T, El- Nemer A, Bashour H. Perceptions about labor companionship at public teaching hospitals in three Arab countries. International Journal of Gynecology & Obstetrics. 2015;129(3):223-6.
11. FIGO. Guideline (2015) Mother—baby friendly birthing facilities. International Journal of Gynecology and Obstetrics. 2015;128:95-9.
12. Morhason-Bello I, et al. Attitude and preferences of Nigerian antenatal women to social support during labor. Journal of Biosocial Science. 2008;40(4):553-62.
13. Michelle M. Client and provider factors associated with companionship during labor and birth in Kigoma Region, TanzaniaBMC pregnancy. 2016.
14. WHO, world health organization, recommendation on companionship during labor and childbirth. The WHO Reproductive Health Library; Geneva:. 2018.
15. Amorim P. presence of a companion of the woman’s choice in the process of parturition: repercussions on obstetric care cogitare enfermagem. 2014;21(4):01-8.
16. Afulani P, Kusi C, Kirumbi L, Walker D. Companionship during facility-based childbirth: results from a mixed-methods study with recently delivered women and providers in Kenya. BMC pregnancy and childbirth. 2018;18(1):150
17. Rala Ntombana JSea. Opinions of laboring women about companionship in labor wards. 2018.

Supporting information

This is the S1.figuer 1 representing benefits of companionship during labor and childbirth

This is the S2 figure 2 the distributions of a reason not to utilize companionship during delivery in Arbaminch town public health facilities

Click here to access/download
Supporting Information
Figure 1.docx
