# Supplementary material for: Utilization of companionship during delivery and associated factors among women who gave birth at Arba Minch town public health facilities, southern Ethiopia
Source: PLoS One. 2020 Oct 2;15(10):e0240239. doi: 10.1371/journal.pone.0240239 (PMC7531811; doi:10.1371/journal.pone.0240239)
Supplement: Attachment — Submitted filename: Response to reviwers biresaw.docx [file pone.0240239.s004.docx]

**Author’s Point-by-Point Response to the Reviewer's and Editors Reports**

**Title**: Utilization of Companionship during delivery and associated factors among women who gave birth at Arba Minch town public health facilities, southern Ethiopia: A cross-sectional study

**Corresponding author**: Biresaw Wassihun /[bireswas@gmail.com](mailto:bireswas@gmail.com)

**Authors**

1. Kassaw Beyene

2. Gebresilasea Gendisha Ukke

3. **Biresaw Wassihun**

**Manscurpuit number:** PONE-D-20-18273

**Journal**: **Plos one**

**Article type:** Research article

**Point by point response to Reviewers and Editors**

First of all, the authors would like to thank Plos one Journal editors and the respective reviewers for reviewing our manuscript and providing the necessary comments to be corrected. As per the comments given, we have made corrections point by point to comment. The authors tried to answer all the issues raised by editorial team and reviewers.

**Point by point response to Editor**

1. Please ensure that your manuscript meets PLOS ONE's style requirements, including those for file naming.""

**Response:** Thank you very much we had apply journal requirement

2. Please include additional information regarding the survey or questionnaire used in the study and ensure that you have provided sufficient details that others could replicate the analyses. For instance, if you developed a questionnaire as part of this study and it is not under a copyright more restrictive than CC-BY, please include a copy, in both the original language and English, as Supporting Information.  If the original language is written in, non-Latin characters, for example Amharic, Chinese, or Korean, please use a file format that ensures these characters are visible

**Response:** ok we will provide All Questioners as additional information upon summation

3. We note that you have indicated that data from this study are available upon request. PLOS only allows data to be available upon request if there are legal or ethical restrictions on sharing data publicly

**Response**: no restriction on data we can attach as supplementary files.

4. Please state whether you validated the questionnaire before testing on study participants. Please provide details regarding the validation group within the methods section.

**Response**: Thank you very much we had validated all tool using Cronbach alpha and we had discussed in detail in the Methods section of the manuscript

5. Thank you for stating the following in the Funding Section of your manuscript:

[Arba Minch University as a requirement for postgraduate studies supports this research
financially.] We note that you have provided funding information that is not currently declared in your Funding Statement. However, funding information should not appear in the Acknowledgments section or other areas of your manuscript. We will only publish funding information present in the Funding Statement section of the online submission form.

**Response**: Thank you very much we had removed it

**Point by point response to Reviewers**

**Question 1:** background is low what is the importance, necessary, gap, and research question.

The structure paragraph is incorrect. Article structure is weak.

**Response 1:** We would like to say thank you very much for your invaluable comments and suggestions. We considered and modified and rewrote again background section based on your constructive issues, coherence, and comprehensibility of the manuscript

**Question 2**: What delivery type with normal vaginal delivery or cesarean delivery?

**Response 2:** those mothers who have a normal vaginal delivery

**Question 3.** exclusion criteria are not completed

**Response 3:** We amend it and corrected it accordingly**.** Those women who are seriously ill and unable to communicate during the data collection period were excluded

**Question 4.** please explain more about validity and reliability of questionnaire

## Response 4: We amend it and corrected it accordingly. After pre-testing the questionnaire, Cronbatch’s Alpha was calculated by using SPSS window version 25.0 to test internal consistency (reliability) of the item and Cronbatch’s Alpha greater than 0.7 was considered as reliable. Data were collected by trained midwives and nurses. During data collection regular supervision was done by the supervisors

**Question 5.** please edit your table? there are a ambitious table with lot of rows

**Response 5:** It was corrected according to your suggestion

**Question 6.** some lines in table have to delete to clarify it.

Response 6. It was corrected accordingly

**Question 7**. discussion is weak and underdeveloped and do not cover all finding such as associated factors.

**Response 7.** In discussion section, correction was made accordingly based on both reviewers comment and suggestion

**Question 8.** the paragraph structure is incorrect.

**Response 8.** correction was made according to you nice comment and suggestion

**Question 9.** Edite your words example South Africa (??)

Response 9. Words correction was made accordingly based on both reviewers comment and suggestion

**Question 10.** The references are incomplete

**Response 10: Correction was made thanks in-depth, for your nice comment**
