# Supplementary material for: Utilization of companionship during delivery and associated factors among women who gave birth at Arba Minch town public health facilities, southern Ethiopia
Source: PLoS One. 2020 Oct 2;15(10):e0240239. doi: 10.1371/journal.pone.0240239 (PMC7531811; doi:10.1371/journal.pone.0240239)
Supplement: Attachment — Submitted filename: Response to reviwers biresaw.docx [file pone.0240239.s005.docx]

**Author’s Point-by-Point Response to the Reviewer's and Editors Reports**

**Title**: Utilization of Companionship during delivery and associated factors among women who gave birth at Arba Minch town public health facilities, southern Ethiopia: A cross-sectional study

**Corresponding author**: Biresaw Wassihun /[bireswas@gmail.com](mailto:bireswas@gmail.com)

**Authors**

1. Kassaw Beyene

2. Gebresilasea Gendisha Ukke

3. **Biresaw Wassihun**

**Manscurpuit number:** PONE-D-20-18273

**Journal**: **Plos one**

**Article type:** Research article

**Point by point response to Reviewers and Editors**

First of all, the authors would like to thank Plos one Journal editors and the respective reviewers for reviewing our manuscript and providing the necessary comments to be corrected. As per the comments given, we have made corrections point by point to comment. The authors tried to answer all the issues raised by editorial team and reviewers.

**Point by point response to Editor**

1. Please ensure that your manuscript meets PLOS ONE's style requirements, including those for file naming.""

**Response:** Thank you very much we had apply journal requirement

2. Please use the space provided to explain your answers to the questions above. You may also include additional comments for the author, including concerns about dual publication, research ethics, or publication ethics. (Please upload your review as an attachment if it exceeds 20,000 characters)

**Response:** ok we will provide

**Point by point response to Reviewers**

**Question** 1. Reviewer #1: I would recommend a round of intensive copy-editing to improve the readibility of the paper. This was a comment made in the previous round of feedback and this challenge persists

**Response**: thank you for your nice comment. All of the comments was edited

**Question** 2. Please state whether you validated the questionnaire before testing on study participants. Please provide details regarding the validation group within the methods section.

**Response**: Thank you very much we had validated all tool using Cronbach alpha . Before actual data collection occurred two-day training was provided for data collectors and the supervisor about techniques of data collection and briefed on each question included in the data collection tool. The pretest was done on 5% (21) of mothers receiving care in a health center that was not included in the study before the actual study period. After pre-testing the questionnaire, Cronbach's Alpha was calculated by using SPSS window version 25.0 to test internal consistency (reliability) of the item, and Cronbach's Alpha greater than 0.7 was considered as reliable. Data were collected by trained midwives and nurses. During data collection, regular supervision was done by the supervisors

**Question 3:** background is low what is the importance, necessary, gap, and research question.

The structure paragraph is incorrect. Article structure is weak.

**Response :** We would like to say thank you very much for your invaluable comments and suggestions. We considered and modified and rewrote again background section based on your constructive issues, coherence, and comprehensibility of the manuscript

**Question 5**: What delivery type with normal vaginal delivery or cesarean delivery?

**Response :** those mothers who have a normal vaginal delivery

**Question 6.** exclusion criteria are not completed

**Response :** We amend it and corrected it accordingly**.** Those women who are seriously ill and unable to communicate during the data collection period were excluded

**Question 7.** please edit your table? there are a ambitious table with lot of rows

**Response :** It was corrected according to your suggestion

**Question 8.** some lines in table have to delete to clarify it.

Response . It was corrected accordingly

**Question 9**. discussion is weak and underdeveloped and do not cover all finding such as associated factors.

**Response .** In discussion section, correction was made accordingly based on both reviewers comment and suggestion

**Question 10.** Edite your words example South Africa (??)

Response 9. Words correction was made accordingly based on both reviewers comment and suggestion
